# Fluency and rule breaking behaviour in the frontal cortex

**DOI:** 10.1016/j.neuropsychologia.2019.107308

**Published:** 2020-02-03

**Authors:** Lisa Cipolotti, Pascal Molenberghs, Juan Dominguez, Nicola Smith, Daniela Smirni, Tianbo Xu, Tim Shallice, Edgar Chan

**Affiliations:** a Department of Neuropsychology, National Hospital for Neurology and Neurosurgery, London, UK; b ISN Psychology, Institute for Social Neuroscience, Melbourne, Australia; c School of Psychology and Mary Mackillop Institute for Health Research, Australian Catholic University, Australia; d Dipartimento di Scienze Psicologiche, Pedagogiche e della Formazione, Università degli Studi di Palermo, Palermo, Italy; e Institute of Neurology, UCL, London, WC1N 3BG, UK; f Institute of Cognitive Neuroscience, University College London, UK; g International School for Advanced Studies (SISSA-ISAS), Trieste, Italy

**Keywords:** Phonemic and design fluency, Prefrontal cortex, Executive, Functions, Rule break errors, Fluid intelligence, Parcel based lesion symptom mapping tract-wise statistical analysis, ATR, Anterior thalamic radiation, CVA, cerebrovascular accident, DF, Design Fluency, FASRB, Phonemic Fluency Rule Break, PFC, prefrontal cortex, GNT, Graded Naming Test, HC, healthy controls, IQ, Intelligence Quotient, LF, Left frontal, LIFG, Left Inferior Frontal Gyrus, LL, left lesion, LMFG, Left Middle Frontal Gyrus, NART, National Adult Reading Test, PLSM, Parcel-based Lesion Symptom Mapping, RAPM, Raven's Advanced Progressive Matrices, RL, Right lesion, TSA, Tract-wise Statistical Analysis, VLSM, Voxel-based lesion symptom mapping

## Abstract

Design (DF) and phonemic fluency tests (FAS; D-KEFS, 2001) are commonly used to investigate voluntary generation. Despite this, several important issues remain poorly investigated. In a sizeable sample of patients with focal left or right frontal lesion we established that voluntary generation performance cannot be accounted for by fluid intelligence. For DF we found patients performed significantly worse than healthy controls (HC) only on the switch condition. However, no significant difference between left and right frontal patients was found. In contrast, left frontal patients were significantly impaired when compared with HC and right frontal patients on FAS. These lateralization findings were complemented, for the first time, by three neuroimaging; investigations. A traditional frontal subgrouping method found significant differences on FAS between patients with or without Left Inferior Frontal Gyrus lesions involving BA 44 and/or 45. Parcel Based Lesion Symptom Mapping (PLSM) found lower scores on FAS were significantly associated with damage to posterior Left Middle Frontal Gyrus. An increase in rule break errors, so far only anecdotally reported, was associated with damage to the left dorsal anterior cingulate and left body of the corpus callosum, supporting the idea that conflict resolution and monitoring impairments may play a role. Tractwise statistical analysis (TSA) revealed that patients with disconnection; in the left anterior thalamic projections, frontal aslant tract, frontal; orbitopolar tract, pons, superior longitudinal fasciculus I and II performed significantly worse than patients without disconnection in these tracts on FAS. In contrast, PLSM and TSA analyses did not reveal any significant relationship between lesion location and performance on the DF switch condition. Overall, these findings suggest DF may have limited utility as a tool in detecting lateralized frontal executive dysfunction, whereas FAS and rule break behavior appears to be linked to a set of well localized left frontal grey matter regions and white matter tracts.

## Highlights

• Performance on fluency tasks is not underpinned by fluid intelligence.
• Design fluency (DF) from the D-KEFS has limited clinical utility.
• Left BA 44/45 and left white matter network underpin Phonemic fluency (FAS).
• FAS rule breaks related to left dorsal ant. cingulate and corpus callosum damage.

## 1 Introduction

Since the original study of Milner (1964), fluency tasks are amongst the most widely used clinical neuropsychological tests for the detection of frontal lobe dysfunction. They involve one key executive function, namely the voluntary generation of non-overlearned responses, which is thought to be implemented by the frontal lobes. One classic verbal generation tasks, which remains very widely used, is phonemic fluency. This task requires the generation of multiple words from a single letter within a given time, typically 1 min. Phonemic fluency appears to be more selective in its loading on frontal lobe processes than a second popular verbal fluency task – semantic fluency – where as many items as possible from a given category need to be generated (Robinson et al., 2012).

The widespread use of verbal fluency tasks has led to an attempt to develop non-verbal analogues. Non-verbal fluency tasks typically involve constrained or free drawing of as many designs as possible in a given time (Jones-Gotman and Milner, 1977). A version of the task developed by Delis and Kaplan (Delis-Kaplan Executive Function System D-KEFS; (Delis et al., 2001), Design Fluency, is probably the most used in standard clinical practice. It has the advantage of having standardized scores that allow comparison with phonemic fluency. In this task subjects are asked to generate drawings that consists of four lines made between filled black dots only, empty dots only or by switching between filled and empty dots (see below for further description). Thus, the final condition of this task involves generation but also switching.

Even though fluency tasks are commonly used in clinical practice and in cognitive neuroscience research, a number of key issues remain poorly investigated and/or the evidence currently available remains inconclusive. The evidence for frontal specialization or lateralization, particularly for non-verbal fluency, is still somewhat inconsistent and sparse. In addition, only a few studies have investigated whether specific frontal sub-regions and related white-matter tracts play a specific role in the different processes involved in fluency tasks. With respect to the behavioural/functional issues, we have previously argued that while most fluency tasks share a common cognitive process, some tasks such as phonemic fluency involve distinct and separable executive processes (Robinson et al., 2012). However, it has also been argued that a single factor –fluid intelligence - can account for all fluency performance (Roca et al., 2010). A further behavioural/functional issue is how errors in performance relate to frontal patients, particularly rule-break errors, in which the patient breaks the known task rules (Andrews et al., 2014). Very little is known regarding the neurocognitive underpinning of rule break errors in fluency tasks, which have been reported anecdotally or combined with other types of errors (e.g. Stuss et al., 1998). Below, we discuss these issues in turn.

Starting with the anatomical questions relating to the issue of specialization and/or lateralization within the PFC, the research on phonemic fluency tasks has been investigated much more than nonverbal fluency. The results however, remain controversial. Thus, many lesion studies have reported reduced phonemic fluency following frontal lobe lesions compared to healthy controls (e.g. Baldo and Shimamura, 1998; Rogers et al., 1998) or to posterior patients (Troyer et al., 1998) and, in some studies, to left posterior patients (e.g. Baldo et al., 2006; Schmidt et al., 2019). However, there are studies reporting equivalent frontal and posterior impairments (Coslett et al., 1991) or a posterior deficit (Vilkki and Holst, 1994). A number of studies reported that verbal fluency is more reduced following left than right frontal lesions, especially for phonemic fluency (Benton, 1968; Milner, 1964). In our previous study, we found greater phonemic fluency impairment in patients with Left Inferior Frontal Gyrus (LIFG) lesions involving BA44/45 compared to frontal patients without LIFG lesions in these two areas (Robinson et al., 2012). Consistent with this, several fMRI studies on healthy controls have supported LIFG involvement in phonemic fluency particularly in BA 44/45 (Costafreda et al., 2006; Heim et al., 2008; Heim et al., 2009; Katzev et al., 2013; Thompson-Schill, D'Esposito, Aguire and Farah, 1997). However, occasional other studies have suggested that reduced phonemic fluency may be due to right frontal or posterior lesions (e.g. Loring et al., 1994; Martin et al., 1990; Perret, 1974). An improvement in phonemic fluency performance in healthy controls following rTMS over the right but not the left frontal cortex has been reported (e.g. Smirni et al., 2017). Hence, some authors have argued that the role of the right hemisphere in phonemic fluency remains controversial (Biesbroek et al., 2016).

Only a handful of studies have adopted VLSM in a large cohort of patients, with lesions not limited to specific cortical regions (e.g. Banerjee et al., 2015; Gläscher et al., 2012; Schmidt et al., 2019). Gläscher et al. (2012) reported that lower performance on the COWAT (Benton et al., 1994) was associated with extensive damage to the left fronto-parietal cortices, anterior prefrontal cortex (PFC) and insula in a large group of patients with stroke, tumours, encephalitis, temporal lobectomy and other focal pathology. Banerjee et al. (2015) found little overlap in the neuroanatomical regions associated with the four expressive measures including phonemic fluency in a group of patients with left glioma. Schmidt et al. (2019) reported in chronic left hemisphere stroke patients that lesions in the pars opercularis (and partly in pars orbitalis) of the inferior frontal gyrus lead to an isolated impairment in phonemic fluency whilst lesions in pars triangularis lead to general impairments in both phonemic and semantic fluency. Notably, in both these two studies right hemisphere lesions were not examined in the VLSM analysis. Given this, it is not possible to draw conclusions about the involvement of right hemispheric structures.

The issue of specialization and/or lateralization within the PFC for nonverbal fluency tasks has, so far, received relatively little attention. The investigations have mainly involved a traditional lesion approach limited by low spatial resolutions, and the results so far are inconclusive. Some studies have found that nonverbal fluency tasks involve frontal brain regions (Jones-Gotman and Milner, 1977; Robinson et al., 2012; Ruff et al., 1994; Tucha et al., 1999). A study by Marin et al. (2017) found poor performance on a design fluency test correlated with lesions in a large distributed network of subcortical, anterior and posterior cortical areas in patients with right hemisphere brain tumours. Other studies found equally severe non-verbal fluency impairments in right and left frontal patients (Tucha et al., 1999). A few studies have investigated the design fluency task of the D-KEFS. They reported no difference between left and right frontal patients (Baldo et al., 2006) or no frontal lateralization in healthy controls using fMRI (Mace et al., 2018). However, in the Baldo et al. (2006) study the sample size (6 left and 5 right frontal patients) was small, therefore leaving lack of power as an important limitation. The Mace et al. (2018) result remains ambiguous since neuroimaging findings in healthy controls may show structures that are not essential to the task.

Our current study's first objective was to reassess the specificity of design and phonemic fluency measures as indicative of involvement, and lateralization, of frontal regions. We used both traditional lesion-mapping and a parcel-based lesion symptom mapping (PSLM) method. To increase power in our analyses, we employed the PSLM approach which uses larger ROIs as a unit and thus significantly reduces the multiple comparison problems (Kimberg et al., 2007).

Our study's second objective was to investigate the white matter connectivity underlying fluency tasks using Tract-wise statistical analysis (TSA; Foulon et al., 2018; Thiebaut de Schotten et al., 2011). Almost all the anatomical approaches to fluency studies have been guided by lesion localization methods. However, other avenues have been advocated for studying brain-behaviour relationships, such as the possible relevance of ‘associationist theories’ (for a review see Catani et al., 2012). These theories rely more heavily on the analyses of network disruption. To the best of our knowledge no such studies have been conducted for nonverbal fluency and only very rarely for verbal fluency tasks. Almairac, Herbet et al. (2015) reported an association between left inferior fronto-occipital fasciculus lesions and poor semantic but not phonemic fluency in 31 patients with left low grade glioma. In another study Li et al. (2017) reported that five left lateralized tracts were significantly correlated with low scores on a Chinese fluency task in 45 stroke patients.

We turn now to the behavioural/functional issues. Our study's third objective was to investigate further the potential role of the medial frontal region and two critical areas of the LIFG (BA44/45) in fluency tasks. As briefly referred to above, fluency tasks have been held to share some processing characteristics. An example is the ability to sustain activation for the duration of the task. Shallice, Stuss, Picton, Alexander and Gillingham (2008) have argued that this process is thought to depend on ‘..energization (cognitive effort) [as] necessary to activate operations not directly triggered in an overlearned fashion by perceptual and motivational inputs..‘. Such energization processes are thought to be localised in the superior medial frontal region (Shallice and Gillingham, 2012; Stuss and Alexander, 2007; Stuss, 2011). In line with this position, a study from our department has previously documented a fluency deficit across 8 verbal and non-verbal fluency tasks in patients with superior medial lesions (Robinson et al., 2012). It has also been argued that phonemic fluency requires specific processes linked to the greater selection demands due to the competition produced by associated stored words that are inappropriately generated by the task rules (e.g., Perret, 1974). Our previous studies suggested that two areas of the LIFG (BA44 and 45) plays a critical role in phonemic fluency when selection demands are high (e.g**.,** Robinson et al., 1998; Robinson et al., 2010; Robinson et al., 2005).

Our study's fourth objective was to establish the extent to which a loss of fluid intelligence can account for fluency impairments. It has been suggested that the frontal cortex carries out a set of general control processes to fulfill the requirements of the task being undertaken, independently of the type of information being processed (e.g. Duncan, 2001). Thus, a large fronto-parietal network, called the *multiple-demand network*, has been shown to be associated with a wide range of cognitive operations in functional imaging work. This network has been proposed to be the seat of fluid intelligence or *g* (e.g. Woolgar et al., 2010). In this approach, the frontal functions involved in any type of fluency task could merely reflect the involvement of the multiple-demand network and its psychological manifestation, fluid intelligence. There is a diversity in the findings reported in the literature. Thus, to the best of our knowledge four studies have supported the idea that impairments in verbal fluency, including phonemic fluency, as well as other executive tests can be explained by impairment in fluid intelligence (Barbey et al., 2012; Barbey et al., 2013; Keifer and Tranel, 2013; Roca et al., 2010). However, one study found no such effect; fluency performance was not explained by fluid intelligence (Robinson et al., 2012).

Our study's fifth and last objective was to further our understanding of the neurocognitive underpinning of rule-break errors in fluency tasks. Behavioral tasks like design and phonemic fluency provides a unique opportunity to examine errors and how they might relate to brain localization/lateralization. Rule-break errors is one specific type of error, where the patient breaks one of the known rules specified by the examiner, even though it is clear the patient understood the rule. Rule-break errors have been reported in frontal patients in a range of executive tasks such as the Multiple Errands (Shallice and Burgess, 1991), the Tower of London (Andrews et al., 2014 see also Köstering et al., 2016) and the Greenwich (Burgess et al., 2000) tests. To date their neurocognitive underpinning and relationship with other executive functions remain largely unknown.

We examined the performance of 53 patients with single, focal, unilateral left or right frontal lesions on the design fluency subtest of the D-KEFS (Delis et al., 2001) and the phonemic fluency task ‘FAS’ (Benton & Hampsher, 1976). We investigated the neural correlates of design and phonemic fluency performance using for the first time in the same population of frontal patients: 1. Traditional lesion frontal subgrouping, comparable to previous published studies (e.g. Robinson et al., 2012; Stuss et al., 2005), 2. Parcel-symptom lesion mapping (PSLM) so that cortical correlates of performance could be examined free from predefined group membership and 3. Tract-wise statistical analysis (TSA) to investigate white-matter correlates of performance.

## 2 Material and methods

### 2.1 Participants

Fifty-three patients with unilateral, focal frontal lobe lesions (left-sided lesion n = 26, right-sided lesion n = 27; see Table 1), resulting from a cerebrovascular accident (CVA), abscess or a tumour resection were prospectively recruited from the National Hospital for Neurology and Neurosurgery (NHNN), Queen Square London as part of a larger study examining cognitive functions of the frontal lobe. The following inclusion criteria were employed: (a) presence of a lesion due to stroke, tumour resection or abscess, (b) lesion entirely confined to the frontal lobe, (c) ability to consent and complete over 75% of neuropsychological and experimental tasks, (d) age between 18 and 80 years, (e) no gross perceptual or language disturbances, i.e. ≥5th cut-off on the Incomplete Letters subtest of the Visual Object Spatial Perception Battery (VOSP; Warrington and James, 1991) and ≥5th %ile on the Graded Naming Test (McKenna and Warrington, 1983) and (f) absence of psychiatric disorders, history of alcohol or substance abuse or previous neurological disorders. Patients underwent a T-1 structural MRI scan as a part of their clinical investigation or for research purposes on either 3 T or 1.5 T Siemen scanners. Lesions were traced and classified by a neurologist who was blind to the study results. We were unable to obtain MRI scans for five of the patients.

**Table 1:** Demographic and cognitive test scores.

	n	Frontal Patients Mean	n	Healthy Controls Mean
Age (years) (SD)	53	46.72 (14.51)	24	50.67 (14.19)
Gender (Male/Female)		27/26		17/7
Education (years) (SD)	34	13.76 (2.68)	24	13.29 (2.35)
NART (SD)	47	110.06 (10.22)	23	109.91 (6.36)
VOSP IL (Correct/20) (SD)	28	19.32 (1.47)	23	19.74 (.45)
GNT (Correct/30) (SD)	34	20.06* (3.80)	23	22.04 (2.92)
Fluid Intelligence SS (SD)	53	10.43 (2.91)	23	11.57 (2.39)

Legend.

**B**old = indicates significant difference between frontal patients and healthy controls.

* = p < 0.05.

NART = National Adult Reading Test.

VOSP IL = Visual Object and Space Perception Battery - Incomplete Letters.

GNT = Graded Naming Test.

SD = Standard Deviation.

SS = Scaled Score.

The aetiologies of the frontal lesions were tumour (n = 41: Left Frontal 20; Right Frontal 21), stroke (n = 11; 5 Left Frontal; 6 Right Frontal) and abscess (n = 1 Left Frontal). Mean years since tumour resection to neuropsychological assessment was 2.75 years (standard deviation = 5.66, range = 0.01–16.89). The mean time between stroke and neuropsychological testing was 7.40 years (standard deviation = 4.98, range = 0.05–10.87). None of these patients have been included in our previous fluency studies. Notably the left and right frontal patients were well matched for aetiology. It should be noted that we have previously shown that the grouping together of frontal patients with different aetiologies for the purposes of examining cognitive variables is methodological justifiable (Cipolotti et al., 2015). In that study, we compared 100 frontal patients with four different types of aetiology on four frontal executive tasks (Letter Fluency-S, Advanced Progressive Matrices, Stroop Colour-Word Test, Trail Making Test Part B). The four groups consisted of one vascular group and three with different types of tumour - high-grade gliomas, low-grade gliomas and meningiomas. The groups did not differ significantly in size or location of lesion. Strong behavioural effects were found of age and premorbid cognitive abilities on performance of the frontal tests. However, on only one test – Trail-Making Part B - was a significant difference between aetiologies obtained when age was partialled out in an ANCOVA. Critically, the significance did not survive Bonferroni correction, as there was no reason to consider Trail-Making, which later research shows not to be specific for frontal lesions (Chan et al., 2015) to be more susceptible to differences in aetiology than the other three tests. The NART did not have a significant effect. Hence, the results of our previous study suggest that combining across vascular and different types of tumour pathologies is not likely to produce a major distortion in the pattern of neuropsychological performance in frontal patients.

In addition, 24 healthy controls (HC) with no history of neurological or psychiatric disorders were included for comparison. The study was approved by The National Hospital for Neurology and Neurosurgery & Institute of Neurology Joint Research Ethics Committee and informed consent was gained from all participants accordingly.

### 2.2 Cognitive investigations

All patients and HCs were assessed on a battery of standardised cognitive tests. All tests were administered and scored in the published standard manner.

#### 2.2.1 Background tests

Premorbid levels of optimal functioning were estimated using the National Adult Reading Test (NART; Nelson, 1982). Perception was assessed using the Incomplete Letters subtest from the VOSP (Warrington and James, 1991), naming ability was assessed using the Graded Naming Test (GNT; McKenna and Warrington, 1983).

#### 2.2.2 Fluid intelligence

Fluid intelligence was assessed using either the well-known Raven's Advanced Progressive Matrices (RAPM; Raven, 1965, n = 27) or the Wechsler Adult Intelligence Scale – Performance IQ (WAIS-PIQ; Wechsler, 1997; n = 26). RAPM is an untimed, relatively culture-free, non-verbal test of abstract reasoning, requiring the selection of the missing piece from a pattern. The total number of correct responses out of 12 items was recorded. Scores were then converted to age-adjusted scaled scores (SS) using available standardized norms. The Wechsler Adult Intelligence Scale – Performance IQ (WAIS-PIQ; Wechsler, 1997) was used as an alternative measure of fluid intelligence as the subtests in the Performance scale has been shown to load heavily on fluid intelligence (Kaufman and Lichtenberger, 1999).

#### 2.2.3 Fluency tasks

##### 2.2.3.1 design fluency

**Fig. 1:**
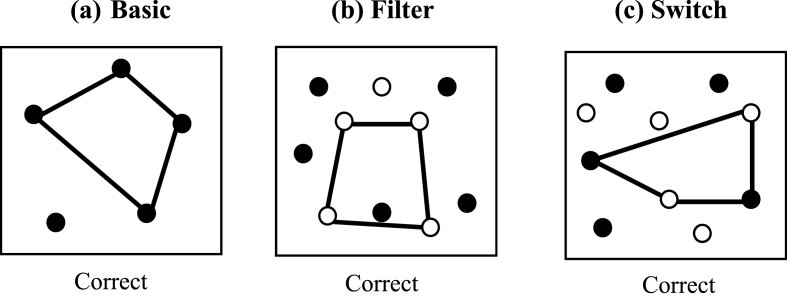
Examples of correct designs for the three conditions of the Design Fluency test (D-KEFS).

Non-verbal fluency was assessed using the Design Fluency subtest of the D-KEFS battery (Delis et al., 2001). This task involves generating as many different abstract designs within 60 s by drawing four straight lines between dot templates. There are three conditions; *basic, filter and switch.* In the *basic* condition, participants were required to draw as many different designs using 4 straight lines between filled black dots (see Fig. 1a). In the *filter* condition, participants were required to draw as many different designs using 4 straight lines between empty dots only (see Fig. 1b). In the *switch* condition, participants were required to draw as many different designs using 4 straight lines switching between filled and empty dots for each line (see Fig. 1c). The three conditions were administered in this order. Participants were given oral and written instructions.

Following the standardized scoring procedure we calculated: the total number of correct designs generated for each condition (design accuracy) and the total number of correct designs across the three conditions. We converted raw scores into age-adjusted scaled scores (SS) using the norms provided by the D-KEFS manual (Delis et al., 2001).

**Fig. 2:**
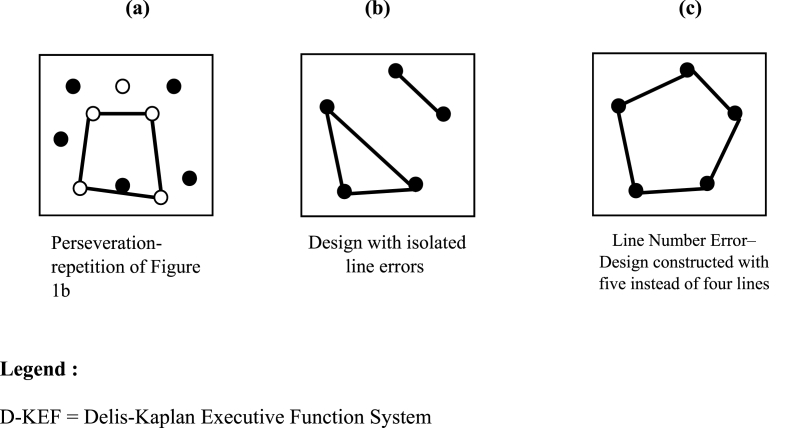
Examples of perseverative (2a) and rule break errors (2 b, 2c).

Incorrect responses (i.e. errors) for all three conditions were firstly calculated following the manual procedure as 1. the total number of designs attempted and 2. the total number of errors. We also investigated 3. the occurrence of repeated designs and 4. set-loss designs. Repeated designs are errors where the same design is drawn two or more times within a condition (i.e. perseverations; see Fig. 2 a). Set loss designs are effectively rule break errors. According to the manual they are: 1. Partially connected line errors i.e. a line that has a dot at one end but no dot at the other; 2. Free floating line errors i.e. designs with a free floating line unconnected by dots; 3. Curved angle errors i.e. designs that form a curved angle of 90° or less between its two end points; 4. Random scribbling errors’ i.e. designs made simply by random scribbling; 5. Isolated line errors i.e. designs with isolated lines i.e. a design that connects two dots, but no other lines are connected to those dots (see Fig. 2, Fig. 6. Line number errors i.e. Designs that contain more or fewer than four lines (see Fig. 2 c).

##### 2.2.3.2 phonemic fluency

We adopted the phonemic fluency test ‘FAS’ (Benton & Hampsher, 1976). This task required participants to orally generate as many words as possible starting with three given letters, F, A and S in 1 min. Participants are told not to produce proper nouns or repeating words. The total number of correct words generated was recorded, excluding errors, and their total raw scores were converted into age-adjusted scaled scores (SS) using the norms from the D-KEFS manual (Delis et al., 2001).Rule break errors (e.g. Samantha for ‘S’) and perseverative responses (any repeated words) were categoried and summed as 1. the number of rule break errors, 2. the number of perseverations and 3. the total number of errors (i.e. rule break plus perseverations).

### 2.3 Lesion mapping

Analyses that revealed significant group differences in fluency performance between frontal patients and healthy controls and between left and right frontal patients were subjected to three neuroimaging investigations: traditional lesion frontal subgrouping, PLSM analyses and TSA.

In the traditional lesion analyses we traced and grouped lesions following an approach based on that of Stuss et al. (2002) and used in several of our previous studies (e.g. Cipolotti et al., 2015; Turner et al., 2007). Specifically, scans were coded each for the presence or absence of lesion in 12 prefrontal areas in each hemisphere (24 in total). The primary lesion had to be >75% of the primary site. These 24 regions were then collapsed and firstly grouped broadly by laterality: left frontal cortex or right frontal cortex. Then lesions were more specifically categorized as Medial or Lateral (Stuss et al., 2005) and either involving or not involving two critical areas Brodmann's areas 44/45 of the Left Inferior Frontal Gyrus (LIFG) known to be implicated in phonemic fluency and verb generation (e.g. Robinson et al., 2012; Thompson-Schill et al., 1998).

PLSM analyses were completed to identify localized frontal damage associated with a lower score on the switch condition of the DF, a lower score on the FAS and a greater number of FASRB using the NiiStat toolbox for Matlab (http://www.nitrc.org/projects/niistat). To increase statistical power, rather than doing voxel-by-voxel analyses, the brain was parcellated in different regions (i.e. ROIs) using the JHU-MNI atlas (Faria et al., 2012). This atlas is implemented in the NiiStat software and contains 185 different ROIs (both grey and white matter regions) basically covering the whole brain. Twenty of these regions are frontal. ROIs that are infrequently damaged will have low statistical power when increasing the number of comparisons. Therefore, to ensure there was enough statistical power to detect a difference, only ROIs where at least five participants had damage were included in the analyses (Sperber and Karnath, 2017).

TSA was performed with the Tractotron software (part of the BCBtoolkit, http://www.brainconnectivitybehaviour.eu). This approach allowed us to investigate the white-matter correlates of impairment in our fluency tasks and in the incidence of rule break errors by mapping the lesion from each patient onto tractography reconstructions of white-matter pathways obtained from a group of healthy controls. For a given lesion, Tractotron provides a probability of disconnection for tracts using recently published white matter tract atlases (Rojkova et al., 2016). When a lesioned voxel overlaps on a white-matter tract with a probability superior to 50% (i.e. above chance), the tract is deemed to be disconnected.

### 2.4 Statistical analysis

Neuropsychological data for the frontal patients and HC was assessed for skewness and kurtosis and tested for normality using the Shapiro-Wilk test.

Independent samples *t*-test or chi-square analyses were conducted for continuous and categorical data respectively to investigate differences between frontal patients and HCs on the demographic variables (age, gender, years of education, NART IQ), and performance on the GNT, VOSP IL and Fluid intelligence.

For the design fluency (DF) and phonemic fluency (FAS) task measures, univariate analysis of variance (ANOVA) was used to examine differences between left frontal patients, right frontal patients and healthy controls. Fluid intelligence and age were entered as covariates. A significant group difference was further examined using post-hoc pair-wise comparisons adjusted with Bonferroni corrections (0.05/3 = p = 0.016). In the frontal patients, we conducted Pearson's partial correlations with one-tailed significance to examine the relationship between DF, FAS and fluid intelligence, with age and time since lesion as covariates. To compare the performance of the left and right frontal patients on the switch condition of the DF and on the FAS, we ran a 2x2 mixed-method ANOVAs with measure as the within-groups factor (DF, FAS), site of damage (left, right) as the between groups factor, and fluid intelligence, age and time since lesion as covariates.

We examined the relationship between DF switch and FAS performance and overall lesion volumes for the left and right frontal patients separately using Pearson's partial correlations, with fluid intelligence, age and time since lesion.

In the traditional lesion approach, using ANOVA, we compared the performance of 1. Patients with Medial and lateral lesions, 2. Patients with Medial lesions with HCs, 3. Patients with LIFG lesions in the critical BA 44/45 areas with patients without lesions in those two LIFG areas. Fluid intelligence, age, time since lesion and lesion volume were all entered as covariates. Partial correlation with one-tailed significance was used to examine the relationship between 1. the extent of the medial lesions 2. the extent of LIFG lesions in BA 44 and 45, and performance on DF switch and FAS. Fluid intelligence, age, time since lesion and lesion volume were all entered as covariates.

**Fig. 3:**
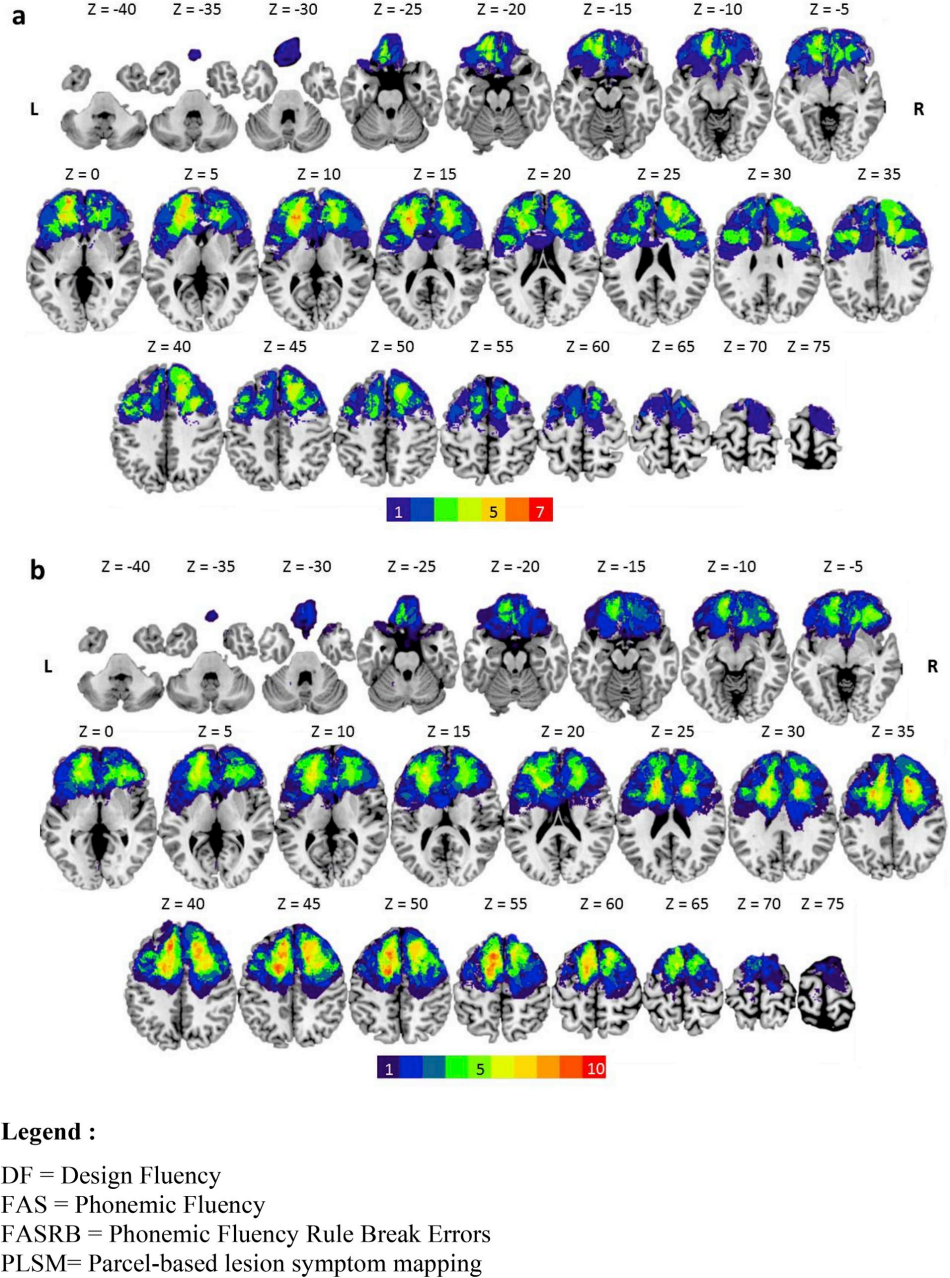
Lesion distribution volume map for all patients used in the PSLM analysis for (a) DF; (b) FAS and FASRB. Results are displayed on transversal slices (numbers indicate MNI coord inates) of the ch2better.nii.gz template in MRIcron (https://www.nitrc.org/projects/mricron). The colour code indicates in how many patients a given voxel was lesioned (ranging from 1 to 10). (For interpretation of the references to colour in this figure legend, the reader is referred to the Web version of this article.)

For the PLSM analyses, Three Freedman-Lane permutations (Winkler et al., 2014) were performed with fluid intelligence, age, time since lesion and lesion volume always entered as nuisance regressors. The lesion distribution map for the switch condition of DF is shown in Fig. 3a and for FAS and FASRB in Fig. 3b. Permutation thresholding (which included 5000 permutations) was used to correct for multiple comparisons and control the family-wise error rate. An alpha of 0.05 was used as the cut-off for significance.

Using TSA, we identified the disconnected white matter tracts across patients. We then used linear regression to compare the performance on DF, FAS and FASRB between spared and disconnected patients, controlling for fluid intelligence, age, time since lesion and lesion volume. To guard against departures from distributional assumptions, results are reported for bootstrapped regressions performed on the basis of 5000 permutations. A tract was included in the analysis only if disconnection was observed in ten or more patients. Significance threshold was set at a p-value of 0.05, corrected for multiple comparisons using the false discovery rate (FDR).

## 3 Results

### 3.1 Demographic results

Frontal patients and HCs were well-matched for age (*t* (75) = 1.115, *p* = 0.269), gender (χ^2^ (n = 77, df = 1) = 2.739, *p* = 0.082), years of education (*t*(56) = -0.697, *p* = 0.489) and NART IQ (*t*(68) = -0.065, *p* = 0.949). Crucially, there was no significant difference between left and right frontal patients on any demographic variable; age (*t* (51) = -0.427, *p* = 0.671), gender (χ^2^ (n = 53, df = 1) = 0.933, *p* = 0.245), years of education *t*(32) = 0.507, *p* = 0.616) and NART IQ (*t*(45) = -1.430, *p* = 0.160). Left and right frontal patients did not differ significantly in the time since the lesion occurred (t (42) = -0.568, p = 0.573) and in lesion volume (t (46) = 0.025, *p* = 0.980).

### 3.2 Cognitive results

There was a significant difference between frontal patients and HC on the GNT (*t*(55) = -2.118, *p = 0*.039); see Table 1). However, there was no significant difference between left and right frontal patients on this test (*t* (32) = 0.094, *p* = 0.925). The lack of difference between left and right frontal patients’ performance on the GNT is consistent with all our previous studies which nevertheless documented lateralized effects for specific executive measures (e.g. Robinson et al., 2012; Cipolotti et al., 2016). Moreover, we have also found no difference on the GNT according to aetiology (Stroke or tumours; Cipolotti et al., 2015). There was no significant difference between frontal patients and HC on the performance of VOSP IL (*t* (32.98) = -1.427, p = 0.163; see Table 1). All patients obtained scores close to ceiling on this task.

#### 3.2.1 Fluid intelligence

There was no significant difference in performance between frontal patients and HC (*t* (74) = -1.409, *p* = 0.163). Only three of the frontal patients performed below the 5th percentile. There was also no significant difference in performance between left and right frontal patients (*t* (51) = -0.643, *p* = 0.523).

#### 3.2.2 Design fluency (DF)

We compared the performance of left and right frontal patients and healthy controls on the Basic, Filter and Switch conditions of DF. In the Basic and Filter conditions, we found no significant differences in performance between frontal patients and HC (F (2, 56) = 0.215, p = 0.807; F (2, 55) = 0.948, p = 0.395 respectively). Hence, these two conditions will not be further analysed in this paper.

On the switch condition, we found a significant main effect of group (F (2, 57) = 5.542, p = 0.007). Post-hoc pairwise comparisons showed that right frontal patients obtained scores significantly lower than HC (p = 0.007). No significant difference was found between left frontal patients compared with HC (p > 0.1). Notably, there was no significant difference between the left and right frontal patients (p > 0.1). Table 2 (A, B C) depicts the mean scaled scores and standard deviations of the left and right frontal patients and HC

**Table 2:** Mean scaled scores and standard deviations for DF and FAS.

	n	LF Patients Mean SS	n	RF Patients Mean SS	n	Healthy Controls Mean SS
	16		18		24	
A) DF Basic Condition (SD)		8.93 ( 3.56)		8.94 ( 3.80)		9.83 ( 3.79)
B) DF Filter Condition (SD)		9.80 ( 2.81)		8.33 ( 3.82)		9.83 ( 3.08)
C) DF Switch Condition (SD)		9.75 ( 2.57)		8.89 ( 2.63)		11.54^a^ *** ( 2.36)
	25		22		23	
D) Phonemic Fluency (SD)		**7.60**^b^*** ( 4.00)		11.77 ( 3.75)		12.17 a *** ( 3.03)

Legend.

LF = Left Frontal.

RF = Right Frontal.

DF = Design Fluency.

FAS = Phonemic Fluency.

SD = Standard Deviation.

SS = Scale Score.

*** = p < 0.01.

Bold indicates significant difference between left frontal and right frontal.

a indicates significant difference between frontal patients and healthy controls.

b indicates significant difference between left and right frontal patients.

##### 3.2.2.1 Design fluency: error analysis

There was no significant difference between left frontal patients, right frontal patients and HCs in the number of total attempted designs (F(2, 54) = 0.219, p = 0.804;see Table 3). In contrast, there was a significant difference in the number of total errors (F(2, 55) = 8.336, p = 0.001), percent design accuracy (F(2, 54) = 8.669, p = 0.001), total repeated designs/perseverations (F(2, 54) = 6.295, p = 0.004) and total rule breaks (F(2, 54) = 3.844, p = 0.028). Critically however, post-hoc pairwise comparisons revealed that both left and right frontal patients made significantly more errors than healthy controls (all p < 0.05), except for total rule break in which only right frontal patients made more errors than healthy controls (p = 0.024). For all error types, there was no significant difference between left and right frontal patients (all p > 0.1).

**Table 3:** Mean Number of Errors on three conditions of the DF.

	Left Frontal Patients Mean No. Errors	Right Frontal Patients Mean No. Errors	Healthy Controls Mean No. Errors
Total Attempted (SD)	28.19 (11.65)	25.75 (7.17)	29.43 (11.31)
Total Error (SD)	5.19 (4.61)	5.82 (3.91)	1.74^a^*** (1.39)
Percentage Accuracy (SD)	83.43 (12.14)	78.25 (14.33)	93.08^a^*** (5.09)
Repeated Designs (SD)	3.81 (3.15)	3.94 (3.34)	1.43^a^*** (1.38)
*Rule Break Errors* (SD)	1.06 (1.81)	1.88 (2.26)	.30^a^*** (.56)

Legend.

DF = Design Fluency.

No. = Number.

SD = Standard Deviation.

*** = p < 0.001.

a indicates significant difference between frontal patients and healthy controls.

We examined the six different subtypes of rule break errors in more detail. Our patient sample did not make any ‘partially connected line errors’ (n = 0), ‘free floating line errors’ (n = 0), ‘curved angle errors’ (n = 0), ‘random scribbling errors’ (n = 0) and made only a very negligible number of ‘designs with isolated line errors’ (n = 6). They did make significantly more ‘line number errors’ (n = 32), i.e. designs constructed of less or more than four lines than HC (χ2 (n = 55, df = 1) = 5.285, p = 0.022). However, there was no significant difference between left and right frontal patients (χ2 (n = 32, df = 1) = 3.205, p = 0.069). Given the lack of significant difference between left and right frontal patients we have not analysed further DF errors.

#### 3.2.3 Phonemic fluency (FAS)

We found a significant main effect of group when we investigated the performance of left and right frontal patients and HC (F (2, 69) = 13.803, p=<0.001). The left frontal patients were significantly impaired when compared to HC and right frontal patients (left frontal patients vs. HC p=<0.001; left frontal patients vs. right frontal patients p=<0.001). There was no significant difference between right frontal patients and healthy controls (p > 0.1; see Table 2).^1^

##### 3.2.3.1 Phonemic fluency: error analysis

There was a significant difference between left frontal patients, right frontal patients and HCs in the number of total errors (F(2, 71) = 5.134, p = 0.008; see Table 4). Breaking down the errors into perseveration or rule break, we found that there was a significant difference in rule breaks (FASRB; F(2, 71) = 5.593, p = 0.006) but not in the number of perseverations (F(2, 71) = 0.381, p = 0.685). Post-hoc pairwise comparison revealed that left frontal patients made significantly more rule breaks (FASRB) than HCs (p = 0.008) and right frontal patients (p = 0.035). The performance of HCs and right frontal patients did not differ significantly (p > 0.1).^2^

**Table 4:** Mean number of errors on FAS.

	Left Frontal Patients Mean No. Errors	Right Frontal Patients Mean No. Errors	Healthy Controls Mean No. Errors
Perseverations (SD)	1.12 (2.22)	.71 (1.12)	.91 (1.04)
Total Error (SD)	**3.52** * (2.86)	1.88 (1.70)	1.61 a* (1.50)
*Rule Break* (SD)	**2.28** b * (2.32)	1.04 (1.20)	.70^a^* (1.11)

Legend.

FAS = Phonemic Fluency.

No. = Number.

* = p < 0.05.

Bold = indicates significant difference between Left and Right frontal patients.

SD = Standard Deviation.

a indicates significant difference between frontal patients and healthy controls.

b indicates significant difference between left and right frontal patients.

### 3.3 Correlations between DF switch, FAS and fluid intelligence

Given that we found a significant group difference only in the Switch condition of the DF (DF switch), we focused only on performance in this condition in all subsequent analyses. We used partial correlation to examine the relationship between performances on the DF switch, FAS and Fluid intelligence, in our frontal patients. We found that there was a significant correlation between FAS and Fluid Intelligence (r = 0.346, p = 0.045) and a trend between DF switch and Fluid intelligence (r = 0.331, p = 0.053). There was no significant relationship between performance on the DF switch and FAS (r = 0.169, p = 0.210).

For the error analyses, given the lack of significant differences between left and right frontal patients, see above, we did not consider the errors measures for the DF task. For FAS, we only focused on rule break errors (FASRB) as these were the only error that revealed a significant difference between left and right frontal patients. There was no significant relationship between the number of rule breaks and Fluid Intelligence (r = 0.177, p = 0.199).

### 3.4 Comparing the performance of left and right frontal patients on DF switch and FAS

**Fig. 4:**
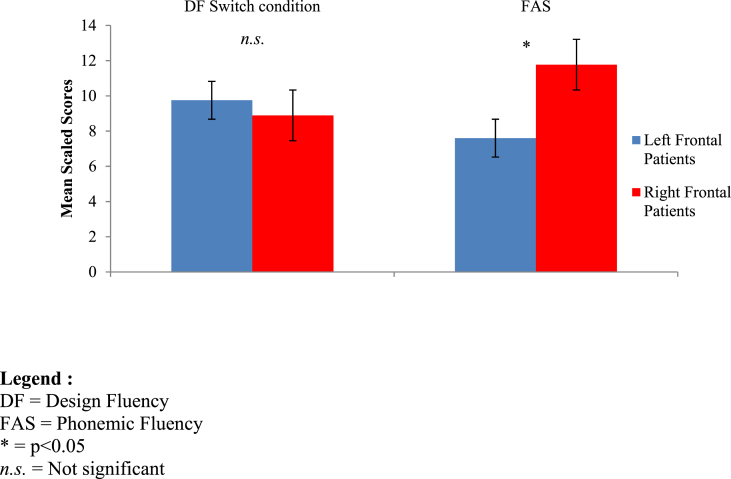
Performance of left and right frontal patients on the DF Switch condition and on the FAS, after accounting for fluid intelligence (Mean Scaled-Scores and standard errors).

We compared the performance on DF switch and FAS for left and right frontal patients, accounting for fluid intelligence, age and time since lesion. We adopted a methodology used in a previous paper (see Cipolotti et al., 2016). We conducted a 2x2 mixed-method ANOVAs with type of measure (DF switch., FAS) as the within-groups factor and site of damage (Left, Right) as the between groups factor. We found a significant main effect for site of damage (F(1, 22) = 4.442, p = 0.047) but not type of measure (F(1, 22) = 1.647, p = 0.213). Importantly, however, we found a significant interaction between the two factors (F (1, 22) = 13.539, p = 0.001). Post-hoc pairwise comparison revealed a significant difference on the performance of the left frontal patients on the DF switch condition and the FAS (p = 0.004). The left frontal patients were impaired in FAS but not on the DF switch condition (see Fig. 4). In contrast, the difference was not significantly different in the performance of the right frontal patients on the switch condition of the DF and the FAS (p > 0.05). These results therefore indicate that the significant interaction between types of measure and site of lesion was driven by the left frontal patients’ impairment on FAS.

### 3.5 Lesion analyses

#### 3.5.1 Traditional frontal subgrouping

For left frontal patients, overall lesion volume was not correlated with DF Switch performance (p = 0.229) but was significantly correlated with FAS (r = −0.458. p = 0.032). For right frontal patients, overall lesion volume was neither correlated with DF Switch nor FAS performance.

For the DF switch condition, we found no significant difference in the performance of medial versus lateral patients (F (1, 14) = 1.382, p = 0.274). There was also no significant difference when we compared medial patients and HC (F (1, 28) = 3.649, p = 0.068; see Table 5b), or between patients with lesions in BA 44/45 of the LIFG (n = 8) or without (n = 21) (F (1, 29) = 0.024, p = 0.877). The extent of medial or LIFG lesion was also not correlated with performance on DF Switch (p > 0.1).

**Table 5:** Mean Scaled Scores for fluency task for specific frontal subgroups.

	Left Lateral Mean SS	Right Lateral Mean SS
DF- Switch condition (SD)	9.50 (2.38)	11.40 (2.30)
FAS (SD)	5.60* (3.58)	13.50 (3.62)

5b) Medial vs HC		
	Medial Mean SS	HC Mean SS
DF- Switch condition (SD)	9.00 (2.35)	11.61 (2.39)
FAS (SD)	8.92* (4.70)	12.27 (3.06)

5c) LIFG vs Non LIFG		
	LIFG Mean SS	Non-LIFG Mean SS
DF- Switch condition (SD)	9.56 (2.40)	9.48 (2.70)
FAS (SD)	6.00* (3.14)	10.94 (4.15)

Legend.

DF = Design Fluency.

FAS = Phonemic Fluency.

Mean SS = Mean Scaled Score.

SD = Standard Deviation.

HC = Healthy Controls.

* = p < 0.05.

**Bold** indicates significant differences between: Left Lateral and Right Lateral on FAS; Medial and Healthy Control on FAS; LIFG patients and non-LIFG patients on FAS.

LIFG = Left Inferior Frontal Gyrus patients.

For FAS, we found no significant difference in the performance of medial versus lateral patients (F (1, 21) = 0.017, p = 0.898). However, we found that medial patients were significantly more impaired than healthy controls (F (1, 35) = 6.502, p = 0.016). When we contrasted patients with lesions in the two critical areas BA44/45 of the LIFG (n = 13) or without (n = 29), we found that the LIFG patients (M = 5.50, SD = 2.68) were more severely impaired than non-LIFG patients (M = 10.45, SD = 3.97) (F (1, 41) = 15.743, p < 0.001). Furthermore, the extent of LIFG lesion was significantly correlated with performance on FAS (r = −0.315, p = 0.029). The relationship was not significant for the extent of Medial lesion (p > 0.1).

#### 3.5.2 Parcel based lesion symptom mapping (PLSM)

PLSM analyses were performed to identify localized brain damage associated with three measures: a lower score on the switch condition of the DF, a lower score on FAS and a greater number of FASRB. These analyses related these three measures to likelihood of damage to regions as defined by the JHU-MNI atlas.

No significant lesion sites were associated with a lower score on the switch condition of the DF.

**Fig. 5:**
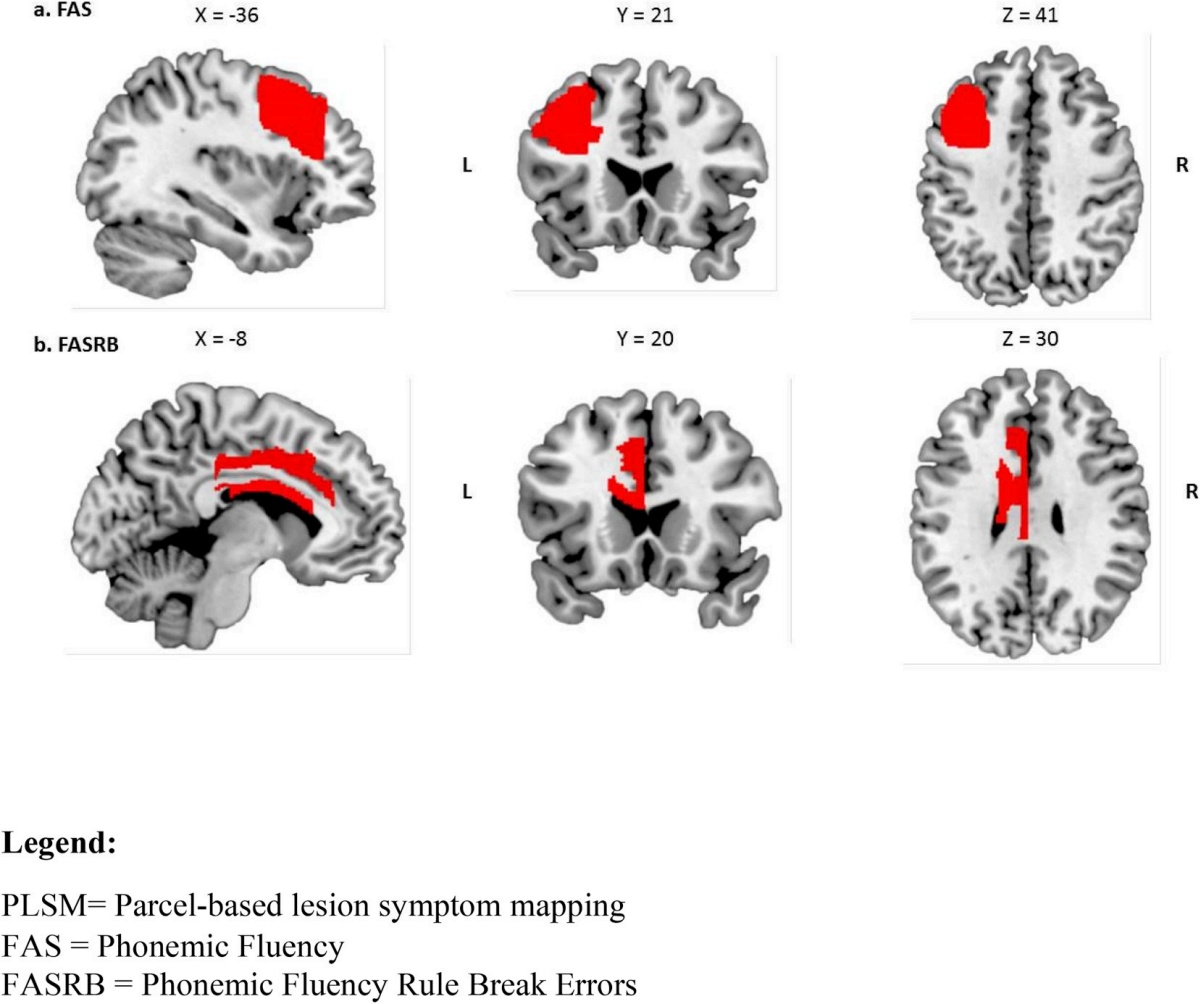
PLSM significant results for (A) FAS and (B) FASRB. Results are displayed on sagittal, coronal and transversal slices (numbers indicate MNI coordinates) of the ch2better.nii.gz template in MRIcron (https://www.nitrc.org/projects/mricron).

In contrast, lower scores on FAS were significantly associated with damage to the posterior segment of the left middle frontal gyrus (Fig. 5a). According to the JHU atlas, this region overlaps with a number of BA areas including areas 5,6,9,46,48 and importantly the upper parts of area 44 and 45. Thus, although the nomenclature is different, all the patients we classified as LIFG patients with our traditional frontal subgrouping approach were effectively encompassed within this left middle frontal gyrus group.

An increased number of FASRB was significantly associated with damage to the left dorsal anterior cingulate and left body of the corpus callosum (Fig. 5b).

The finding from our PLSM analyses indicating that lower scores on FAS was significantly associated with damage to posterior left middle frontal gyrus appears somewhat discrepant from the traditional frontal subgrouping lesion findings implicating the LIFG. However, as already stated above, the LMFG, as identified by the JHU atlas, contains a large number of Brodmann areas. Critically, it includes the upper parts of BA 44 and 45, two areas that our traditional frontal subgrouping classifies as LIFG areas.

In an attempt to clarify further the contribution of the LMFG and LIFG we identified the patients whose LMFG lesions, as identified by the JHU atlas, did not involve either BA44 or BA45, as identified by our frontal subgroupings. When we contrasted these 7 LMFG patients without BA 44 or BA45 lesions with frontal patients without LMFG lesions (Non-LMFG n = 25), we found no significant difference in the FAS performance (LMFG without BA44 or BA45 lesions: M = 9.43, SD = 5.44; Non-LMFG: M = 11.36, SD = 3.74; (t(30) = -1.09, p = 0.284). This finding suggests that it is the presence of lesion in BA 44/45 in patients with LMFG lesion that potentially contributes to their poor performance on FAS. Notably, the mean performance on FAS of the LMFG patients without BA44/45 lesions was higher (M = 9.43, SD = 5.44) than the mean performance of the LMFG patients with lesions primarily involving BA44/45, as identified by our frontal subgroupings (M = 6.00, SD = 3.14).

#### 3.5.3 Tract-wise statistical analysis (TSA)

TSA found no significant relationship in the performance on the DF switch between patients with disconnected or spared left or right frontal tracts (FDR corrected *p* > 0.05).

**Fig. 6:**
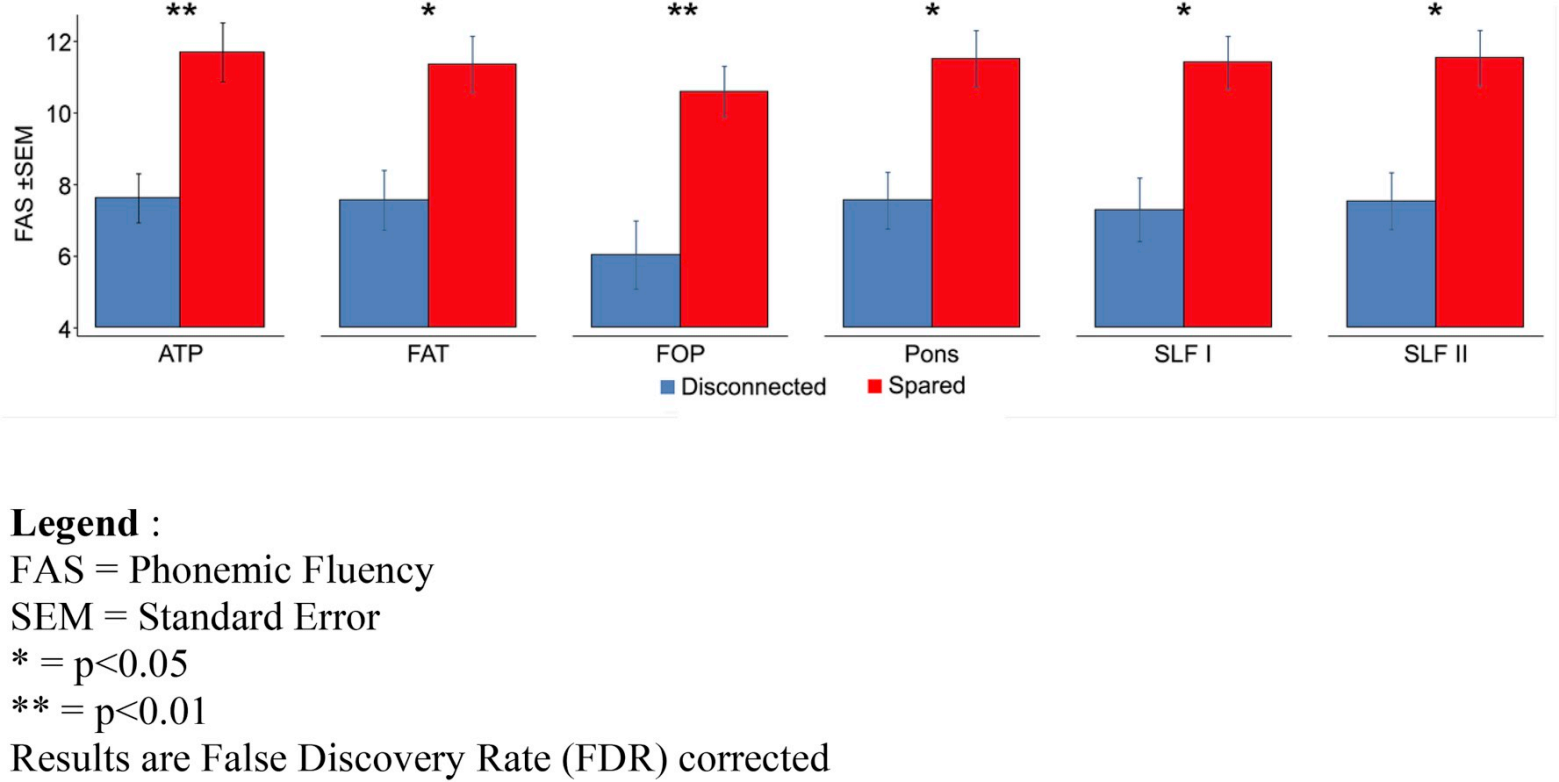
TSA. Bar graphs depicting the significant difference in FAS performance between frontal patients with and without a disconnection in the left anterior thalamic projections (ATP), frontal aslant tract (FAT), frontal orbitopolar (FOP) tract, pons and superior longitudinal fasciculi I and II (SLF I and II).

In contrast, for the FAS performance TSA revealed that patients with a disconnection in the left anterior thalamic projections, frontal aslant tract, frontal orbitopolar tract, pons, superior longitudinal fasciculus I and II performed significantly worse than patients without disconnection in these tracts (FDR corrected, *p* < 0.05; see Fig. 6). Moreover, we found no difference between the performances of patients with disconnected or spared right frontal tracts (FDR corrected, *p* > 0.05).

We found no significant relationship in the number of FASRB between patients with disconnected or spared left or right frontal tracts (FDR corrected *p* > 0.05).

## 4 Discussion

We investigated a number of anatomical and behavioural aspects of the performance on the Design fluency test (DF) from the D-KEFS (Delis et al., 2001) and on the FAS (phonemic fluency) in a sample of patients with focal frontal lesions. Importantly, for the first time, in the same population of frontal patients, we complemented the behavioral measures with a range of localization procedures. Thus we used traditional frontal lesion subgrouping, PLSM, and TSA to investigate the cerebral localization underlying fluency and rule break errors.

The Discussion addresses a number of topics. First we consider whether fluency performance can be entirely reduced to fluid intelligence. The contribution of fluid intelligence was unknown for DF and poorly understood for FAS. Having held that such reduction is not possible, we secondly consider DF performance, whether it has a major underlying frontal component, and if so whether this can be lateralized and localized, and if it mirrors in the non-verbal realm, the frontal components of FAS. Then, the lateralization and, in particular, the localization within the left frontal lobe of processing systems and pathways underlying FAS performance is considered. Finally, using the same methods, we provide an analysis of rule break errors that have only been anecdotally reported in the literature. Our results further our understanding of the functional organization of the frontal cortex and provide some novel insight into frontal lobe impairments and associated regions of damage.

We found that our frontal patients were significantly impaired when compared with HC on the FAS and on only one of the DF conditions, namely the *switch condition*. The difference in performance between frontal patients and HC on the fluency tasks remained significant, even when fluid intelligence was taken into account. These findings indicate that performance on the DF switch and FAS cannot be entirely accounted for by a reduction in fluid intelligence, consistent with our findings from another sample of frontal patients tested with a different non-verbal fluency task (Robinson et al., 2012). Fluency represents another example of ‘executive’ tasks for which impairment cannot be accounted for entirely by fluid intelligence (see for further examples Proverb Interpretation, Stroop, Hayling and Cognitive Estimates; Murphy, Shallice, Robinson, MacPherson and Turner, 2013; Cipolotti et al., 2016; Cipolotti et al., 2017).

Our results are, however, at odds with previous studies that have suggested fluid intelligence could account for the frontal patients' phonemic fluency impairments (Barbey et al., 2012; 2013; Keifer and Tranel, 2013; Roca et al., 2010). These contrasting results could possibly due to the different measures used for fluid intelligence. Thus, we used Raven's Progressive Matrices and WAIS III Performance IQ as our main measure of fluid intelligence, while others have used WAIS FSIQ (e.g. Barbey et al., 2012) or the Culture Fair test (e.g. Roca et al., 2010). However, probably more critically, Roca and colleagues included only 7 patients with lesions in the left lateral frontal areas and only 2 patients had a LIFG lesion. Thus, patients with lesions in an area known to contribute to FAS performance were clearly under-represented. Moreover, the patients reported by Barbey et al. (2012) were veterans with penetrating, focal traumatic brain injury that can lead to diffuse injuries. In Keifer and Tranel’s (2013) study, only a relatively small number of patients were included in each lesion group.

To date, very little is known regarding whether the DF test from the D-KEFS is a reliable test for detecting frontal brain impairment and if a frontal lateralization effect is present. Our findings suggests that the *basic and filter conditions of the DF* are not reliable for detecting brain impairment as there was no difference between the performance of frontal patients and HC, once the effects of fluid intelligence and age were partialled out. In contrast, the DF switch condition, total number of errors, the percent of design accuracy, total repeated designs and rule break errors, can be considered reliable for detecting brain impairment. Critically however, performance on the DF switch did not distinguish between left and right frontal patients (see for similar findings Baldo et al., 2001). Nor, does examining lateralization of frontal lesions produce significant differences in total number of errors, the percent of design accuracy, total repeated designs and rule break errors.

Our traditional frontal subgrouping analysis for the DF switch also did not produce significant differences in the performance of medial versus lateral patients or medial versus healthy controls, and there was no correlation between the extent of medial or lateral lesion. Moreover, our PLSM analysis failed to identify any significant frontal lesion site associated with poor performance on the DF switch. The TSA also found no significant relationship between patients with disconnected or spared frontal tracts. These two analyses have not been reported before for this task. They further corroborate the notion that performance on the DF switch does not localise to any specific frontal region.

The lack of a localization effect for the DF switch may be due to the fact that this is not an entirely nonverbal test as it contains the instruction to make four lines and so it involves counting. Moreover, this test has a task-switch as well as a fluency component. Although our findings show a lack of localization effect we are not suggesting that this task does not require frontal lobe involvement. It may well be that the DF has a number of components which localise differently. In this respect the results on DF are difficult to interpret. In our view the most likely possibility is that performance on this task relies on a distributed network involving several subsystems in the left and right frontal regions. Functional imaging has been one useful way of elucidating underlying brain networks involved in performing nonverbal fluency tasks. In two separate studies different right and left frontal areas were activated according to the type of strategy used. Visuo-spatial strategy was associated with bilateral frontal areas while a mixture of visuo-spatial and verbal strategies was associated with left DLPFC (Elfgren and Risberg, 1998) or posterior areas (Suchy et al., 2010). Moreover, in a lesion study of rotation ability, a prototypical visuo-spatial task, along with a right parietal effect, a left PFC effect was also found, but not a right PFC one (Buiatti et al., 2011). Another possibility as to why we did not find a significant effect may be due to the brain atlas that we applied in our PLSM analyses. We chose this method over VLSM to increase power in our analyses and in an attempt to reduce the multiple comparison problems (Kimberg et al., 2007). However, one of the limitations of our approach is that it requires applying boundaries to define our different brain regions. Thus, if the area of interest overlaps two regions, we may unintentionally reduce the likelihood of detecting the effect.

Most critically, despite the caveats just discussed, the presence of significant lateralization and localization findings for the FAS measure makes it unlikely that the regions involved in non-verbal fluency directly mirror what is found for verbal fluency. Furthermore, the null findings from our behavioural analyses, traditional subgrouping analyses and TSA provide converging evidence regarding the lack of localization and lateralization effects for the DF switch. Again, as we will discuss shortly this differs greatly from effects found with the FAS measure. DF does not mirror what is found with verbal fluency.

From a clinical perspective, DF switch, total number of errors, the percent of design accuracy, total repeated designs and rule break errors, can offer insights regarding a patient's cognitive abilities. However, our findings suggest it may have limited utility as a tool in detecting lateralized frontal executive dysfunction specifically as task performance most likely depends upon a set of cognitive processes, some of which require left and some right frontal brain regions. As such, caution should be used when drawing conclusions from the DF switch performance alone. Further work is needed to establish whether the current findings also extend to the various alternative versions of nonverbal fluency tasks (e.g. Jones- Gotman & Milner, 1977).

Turning to the FAS, we found evidence for lateralization in its functional organization. Left frontal patients were significantly impaired when compared to both HC's and right frontal patients on overall performance, total and rule break errors but not on perseverative errors. Moreover, after accounting for fluid intelligence, age and time since lesion, we found a significant interaction between the performance of the left frontal patients on FAS and on DF switch. The left frontal patients were impaired in FAS but not on DF switch. These findings are in broad agreement with previous literature suggesting a critical role of the left frontal lobes for FAS (e.g. Benton, 1968; Milner, 1964; Robinson et al., 2012; Schmidt et al., 2019).

These lateralization findings were complemented and extended by the results of our three neuroimaging investigations. Our traditional frontal subgrouping method, previously adopted in the literature (for a review, see Stuss, 2011), indicated significant differences in FAS performance between the medial frontal patients and HC and between patients with or without LIFG lesion in two critical areas BA44/45. The current results are in broad agreement with our previous findings (Robinson et al., 2012). In this respect they represent an important instance of independent replication in an area where traditionally there has been a paucity of replication and an abundance of contradictory findings (e.g. Shallice, 1982, 2015). In addition, the present study also found a significant relationship between the extent of LIFG lesion, but not medial, and performance on FAS. This suggests that both the location and the extent of the LIFG lesion may be critical.

Our PLSM analysis found that lower scores on FAS were significantly associated with damage to posterior LMFG. This finding is in keeping with the work of Biesbroek et al. (2016). They found in stroke patients, that phonemic and semantic fluency share large overlapping anatomical correlates involving left medial and inferior frontal areas as well as the left precentral gyri, rolandic operculum, insula and putamen. Critically, in their study, the LMFG was associated with poor phonemic but not semantic fluency. Our PLSM findings indicating that lower scores on FAS were significantly associated with damage to posterior LMFG appears discrepant from our earlier frontal subgrouping lesion findings implicating the LIFG. Our additional analysis suggested that patients classified as having LMFG lesions according to PLSM also included patients with lesions involving BA 44/45 of the LIFG. When we contrasted the performance of patients with LMFG without lesions in BA 44 or 45 with non LMFG patients we found no significant difference in performance on FAS. Qualitatively, we noted that patients with LIFG lesions involving BA 44/45 obtained the lowest scores on FAS when compared with LMFG and non-LMFG patients. This tentatively suggests that it is the presence of a lesion involving BA 44/45 of the LIFG that contributes to the poor performance on FAS. This finding further supports the notion that this region plays a critical role in verbal generation tasks requiring greater selection demands due to the competition produced by associated stored words (e.g. Heim et al., 2008, 2009; Katzev et al., 2013; Robinson et al., 1998, 2005; 2010, 2012; Schnur et al., 2009; Thompson-Schill et al., 1997).

To the best of our knowledge, there has been no attempt in the literature so far to reconcile in the same study the findings obtained with an approach based on categorically grouping patients on the basis of a common lesion with findings obtained from a lesion symptom mapping approach. Of course, one needs to be cautious when drawing conclusions concerning the exact localization of critical sites for phonemic fluency using techniques such as PLSM (see the concerns raised regarding the limitations of VLSM, another example of mass-univariate approach to human brain mapping, e.g. Mah et al., 2014, 2015; Nachev, 2015; Xu et al., 2018). Indeed, in the last few years, several studies have promoted the use of multivariate decoding and computational modelling of data (Herbet2015; Smith et al., 2013; Xu et al., 2018) which theoretically, offer higher sensitivity for modelling lesion-behavioural predictions. However, this is confounded by the discrepancy between sample size and the number of neuroimaging features (e.g. Chen, 2009; Bellman, 2015; Indyk and Motwani, 1998; Lee and Yoon, 2017; Nasrabadi, 2007; Sinha et al., 2009; Verleysen and François, 2005). PLSM and TSA represent a midway solution as they are coarser than voxel-wise analyses. These approaches, we suggest, should be expected to be more robust to spatial biases, as parcels and tracts are more likely, given their size, to encompass the displacement of the critical locus. This is, however, a question that needs to be resolved empirically. The emphasis here is not to exactly map a symptom with voxel-level precision but to a larger anatomical or functional unit. Ideally, if one was able to collect a much larger number of patients, in the order of hundreds, it would be possible to switch from parcel/tract-wise to multivariate voxel-wise approach.

TSA allowed us to capture the relationship between frontal white matter damage and observed FAS impairment. We found that patients with a disconnection in the superior longitudinal fasciculus I and II, frontal aslant tract, frontal orbitopolar tract, left anterior thalamic projections and pons performed significantly worse than patients without disconnection in these tracts on FAS. Interestingly, we found no difference in the performance between patients with disconnected and spared right frontal tracts. These findings suggest that the complex set of executive functions involved in FAS performance rely on widely distributed left lateralized networks sub-served by long association and commissural white matter pathways (see for a similar point Makris et al., 2005; Petrides and Pandya, 2006; Schmahmann and Pandya, 2007). They support the broader notion that regions of high tract overlap represent critical anatomical areas that may have a negative impact on cognitive functions such as the voluntary generation of non-overlearned responses (e.g. Corbetta et al., 2015; Griffis et al., 2017).

To our knowledge, very few studies have previously investigated the relationship between white matter pathways and verbal fluency impairment (Almairac et al., 2015; Li et al., 2017; see for examples for other cognitive functions Forkel and Catani, 2018; Ivanova et al., 2018; Nakajima et al., 2018; Toba et al., 2017). It remains an open question whether Li et al. (2017)'s findings which correlated tract damage with scores on a Chinese fluency task, can generalize across languages. Notably, the Almairac et al. (2015) study did not include patients with right frontal lesions. This somewhat limits the significance of their reported left tract lateralization. A similar limitation also applies to the Griffis et al. (2017) study which investigated white matter bottlenecks or ‘structural weak points’ using deterministic tractography in 43 patients who all had chronic left hemisphere stroke. The authors reported that damage to a posterior bottleneck region underlying the posterior temporal lobe, including fibres associated with both dorsal arcuate fasciculus, ventral inferior longitudinal fasciculus and inferior fronto-occipital fasciculus (IFOF) was critical for performance on verbal fluency (a composite score of semantic and phonemic fluency), as well as other language measures. However, damage to the anterior bottleneck including left anterior thalamic radiation, uncinate fasciculus and IFOF predicted deficits in verbal fluency.

Our TSA findings suggest that mapping the white matter damage in PFC patients can expand understanding of the critical lesions underlying observed behavioral deficits on the FAS task. It also has the potential to increase knowledge of the organization of the networks sub-serving phonemic fluency. Previous research has linked the left anterior thalamic projections (Nishio et al., 2011**)** and the superior longitudinal fasciculus (Bernal et al., 2010; Kamali et al., 2014; for a review, see Dick et al., 2014) to a variety of language functions (e.g. fluency, phonemic paraphasias, syntactic processing and language learning), although a link with executive functions has not been consistently reported.

Future studies are needed to investigate further the nature of the left frontal tract disconnections we identified as critical. It may well be that non-frontal lesions also damage our reported tracts and this in turn may result in poor FAS performance. Similarly, future research is needed to elucidate the relationship between our reported white matter disconnection and the ‘lesion load’, namely the proportion of the tract that has been destroyed (see for similar discussion Hope et al., 2016). It is also important to stress that TSA is an indirect measure of damage to white matter tracts, as it relies solely on structural, T_1_-weighted data. While this greatly expands what we can learn about the impact of lesions to the brain from a single imaging modality, studies that reconstruct a tractogram directly from diffusion-weighted patient data are essential.

Lastly, we found that our left frontal patients made a significant number of FASRB errors. Aldermanet al, (2003) reported in a sample of mainly traumatic brain injury patients (78% of the cases) that rule break behavior was associated with some factors from the Dysexecutive questionnaire (DEX-S; Burgess et al., 1996). Difficulties in formulating goal oriented plans were specifically related to rule break measures as was the performance on the action program test of the Behavioral Assessment of the Dysexecutive Syndrome Battery (Wilson et al., 1997) assumed to require among various functions a reasoning component (see also Klosowska, 1976).

In our view, the increased number of FASRB errors made by our left frontal patients is difficult to explain in terms of a general failure to shape performance by task goals or impaired reasoning. General task goal maintenance or reasoning is required also by other tasks, such as for example, our demanding fluid intelligence tests. Hence, according to this view, task goal or reasoning impairments should lead to deficits also in these tasks. However, we found that our frontal patients were not significantly impaired on the fluid intelligence tests when compared to healthy controls, nor did we find a difference between left and right frontal patients. Moreover, we found no significant relationship between worse performance on the fluid intelligence tests and the high number of FASRB. Hence, it seems that a general failure in control of behaviour by task goals or impaired reasoning cannot be easily reconciled with the left lateralized effect for FASRB errors we documented.

Our PLSM analysis documented, for the first time that an increased number of FASRB was significantly associated with damage to the left dorsal anterior cingulate and left body of the corpus callosum. Both areas have been implicated as critical for executive functions (Bettcher et al., 2016). The authors examined the structural relationship between indices of executive functions (i.e. shifting/inhibition and updating/working memory), prefrontal, non-frontal lobar volumes and global grey matter in a cohort of healthy older adults. They reported that higher corpus callosum and cingulate (dorsal) fractional anisotrophy predicted better executive functions, independent of global grey matter atrophy. Moreover, our reported critical areas for FASRB are in broad accord with the suggestion of Burgess et al. (2000). The authors reported that on the Greenwich test, damage to the left frontal cortex, including the posterior cingulate area and left medial regions such as BA 8, 9 and 10 resulted in an overall poor task performance, using a score that penalizes rule break behavior. However, our PLSM findings for FASRB are in contrast with the recently reported finding of Zhang et al. (2016) who reported a lack of a significant correlation between the number of rule-break errors and any brain region in a healthy sample. It is possible that the Zhang and colleagues result may be due to the very low frequency of rule-breaking behavior observed in their sample.

We would argue that our reported association between FASRB and damage to the left dorsal anterior cingulate and left body of the corpus callosum fits with the idea that conflict resolution and monitoring impairments may play a role for this type of errors. Hence, the anterior cingulate region has been previously involved in conflict monitoring (e.g. Botvinick, 2007; Carter et al., 2001; Kerns et al., 2004; Sheth et al., 2012), error processing (Dehaene et al., 1994; Gehring and Knight, 2000; Kiehl et al., 2000) and urgent inhibition over faster or more automatic behaviors (e.g. Garavan et al., 2002). Köstering et al. (2016) investigated rule break errors on the Tower of London task in a large variety of neurological patients (60 Strokes, 51 Parkinson's, 29 Mildly Cognitively Impaired patients) and 75 HC. They suggested that deficits in self-monitoring, as well as other cognitive impairments, may underpin rule break errors. Such a difficulty in self-monitoring may itself derive from an even more basic process – energization of non-automatic processing – which would heavily involve the anterior cingulate (e.g. Shallice and Gillingham, 2012).

Our TSA found no significant relationship in the number of FASRBs between patients with disconnected or spared left or right frontal tracts. This may be due to the fact that FASRB is a somewhat less sensitive measure than FAS. This is illustrated by the much more robust difference between left and right frontal patients in the FAS score relative to the number of FASRB errors. This reduced sensitivity, in turn, may have decreased the possibility of being able to identify critical white matter tract disconnections.

One general caveat of our approach is that in order to obtain a sizeable sample of focal, unilateral, frontal lesions for our investigations, we grouped together frontal patients with different aetiologies (see for similar approach Aridan et al., 2019; Aron et al., 2004; Gläscher et al., 2012; Roca et al., 2010; Stamenova et al., 2017; Stuss et al., 2005; Thompson –Schill et al., 1998; Urbanski et al., 2016). We previously demonstrated that combining vascular and different types of tumour pathologies is not likely to produce a major distortion in the pattern of neuropsychological performance in frontal patients (Cipolotti et al., 2015). It remains a practical necessity to mix aetiologies in order to obtain large groups of patients to investigate behavioural/functional issues, even in a major neurological hospital, such as the NHNN with a department of neuropsychology that oversee the largest number of patients in the UK (over 5000 patients per year). This is a well-recognised problem in neuropsychology. For example, Andres and Van der Linden (2001) noted that in order to obtain a group of 13 frontal patients (mixed aetiology including TBI), “… took four years and involved five large hospitals…“). To attempt to eliminate all possible artefacts would make it impossible in practice to obtain useful results, especially if one aims to have as the critical group, patients whose lesions are restricted to frontal cortex. In our current study to reduce the danger of artefactual conclusions linked to different types of pathologies the right and left frontal groups contained similar number of stroke and tumours respectively.

Of course, any attempt to combine across patients in neuropsychology group studies is liable to suffer from potential confounds. Indeed, some studies favour use of a single aetiology, normally stroke (e.g. Baldo et al., 2006; Campanella et al., 2016; Sperber and Karnath, 2017; Varjačić et al., 2018). Arguments can be presented in favour of each alternative. Moreover, there is not just a single line that can be drawn. Does, for instance, one include both infarcts and haemorrhages under stroke for the single aetiology position? Does one include head injury for the mixed aetiology position? To the best of our knowledge there are no definitive review articles addressing these issues which comes down definitively one way or the other. In the absence of a consensus in the field, at least we have attempted to show that the grossest dangers of using the mixed aetiology approach, namely that some should be much more severe than others in their effects, does not hold (Cipolotti et al., 2015).

In conclusion, our study suggests that performance on DF and FAS is not underpinned by a general fluid intelligence process. Two of the three conditions *basic and filter conditions* of DF are not reliable for detecting brain impairment. The switch condition*,* the total number of errors, the percent of design accuracy, total repeated designs and rule break errors are useful measures to differentiate performance between frontal patients and healthy controls. However, they do not allow one to detect the lateralization of frontal executive dysfunction. Hence, the DF test from the D-KEFS is of limited utility as a clinical tool.

In contrast, a set of well localised left frontal regions and disconnection of left frontal tracts appear to play a crucial role in performance on FAS and rule break behaviour. Hence, both measures are clinically relevant when assessing executive functions in brain damage patients. The adoption of different neuroimaging techniques assessing the contribution of cortical areas and white matters tracts appears to be a fruitful approach in furthering our understanding of the neurocognitive architecture underpinning the complex executive processes involved in fluency tasks (see Special Issue: Lesion and Brain Mapping (Chechlacz et al., 2018 for further discussion).

## Funding

This work was supported by the Welcome Trust Grant (089231/A/09/Z). This work was undertaken at UCLH/UCL, which received a proportion of funding from the Department of Health's National Institute for Health Research Biomedical Research Centre's funding scheme. P. M. was supported by a Heart Foundation Future Leader Fellowship (1000458).

## CRediT authorship contribution statement

**Lisa Cipolotti:** Investigation, Resources, Data curation, Writing - original draft, Writing - review & editing, Visualization, Supervision, Project administration. **Pascal Molenberghs:** Resources, Data curation, Writing - review & editing, Visualization. **Juan Dominguez:** Resources, Data curation, Writing - review & editing, Visualization. **Nicola Smith:** Investigation, Data curation, Writing - original draft, Writing - review & editing, Visualization, Project administration. **Daniela Smirni:** Investigation, Resources, Data curation, Writing - review & editing. **Tianbo Xu:** Investigation, Resources, Data curation, Writing - review & editing. **Tim Shallice:** Writing - original draft, Writing - review & editing, Supervision. **Edgar Chan:** Investigation, Resources, Data curation, Writing - review & editing, Visualization, Supervision, Project administration.

## Footnotes

1 The same analyses on phonemic fluency performance were conducted for patients who had WAIS-PIQ (*n* = 40) or RAPM (*n* = 27) separately to ensure that the effect of fluid intelligence was not masked by collapsing the two measures. The pattern of performance was unchanged.

2 The same analyses on phonemic fluency errors were conducted for patients who had WAIS-PIQ (*n* = 40) or RAPM (*n* = 27) separately to ensure that the effect of fluid intelligence was not masked by collapsing the two measures. The pattern of performance was unchanged.
